# Fifteen years of pediatric immune thrombocytopenia in a national cohort: chronicity, diagnostic challenges, and treatment patterns—single center experience

**DOI:** 10.3389/fped.2026.1864433

**Published:** 2026-06-11

**Authors:** Katja Pregeljc, Elena Favaretto, Federico Verzegnassi, Barbara Faganel Kotnik

**Affiliations:** 1 Faculty of Medicine, University of Ljubljana, Ljubljana, Slovenia; 2 University Medical Center Ljubljana, Ljubljana, Slovenia; 3 Department of Pediatrics, Ca’ Foncello Hospital, Treviso, Italy; 4 Oncohematology Unit, Institute for Maternal and Child Health IRCCS Burlo Garofolo, Trieste, Italy; 5 Department of Hematology and Oncology, University Children’s Hospital, University Medical Center Ljubljana, Ljubljana, Slovenia

**Keywords:** alternative diagnoses, chronicity, complications, immune thrombocytopenia, pediatrics, watch-and-wait management

## Abstract

**Background:**

The majority of children with primary immune thrombocytopenia (ITP) follow a benign, self-limiting course. However, early identification of children at risk for chronic disease, timely recognition of alternative diagnoses that may mimic ITP at onset, and selection of patients for observation alone remain important clinical challenges.

**Methods:**

This retrospective single-center study included patients aged ≤18 years who were evaluated for ITP between 2009 and 2024. Demographic, clinical, laboratory, and therapeutic data were collected at diagnosis and during follow-up. Factors associated with chronic ITP were assessed using logistic regression, providing odds ratios with 95% confidence intervals. Kaplan–Meier analysis was used to describe time to first bleeding complication according to initial management.

**Results:**

A total of 271 patients were included (43.5% female; median age 4 years). Of these, 240 were ultimately diagnosed with ITP, while 31 received an alternative final diagnosis. In multivariate logistic regression, chronic ITP was independently associated with older age (OR 1.08; *p* = 0.017), absence of a preceding infection or vaccination (OR 0.45; *p* = 0.029), and higher platelet count at diagnosis (OR 1.03, *p* < 0.0001). Compared with patients with final ITP, those with alternative causes of thrombocytopenia were older (*p* = 0.034), had lower bleeding grades (*p* = 0.037), higher MCV, and lower total leukocyte, lymphocyte, monocyte, and eosinophil counts at onset (all *p* < 0.05). Patients receiving upfront pharmacological therapy were younger and presented with higher bleeding grades and lower platelet counts than those managed with observation. Bleeding complications were more frequent with upfront pharmacological therapy, whereas those in the watch-and-wait group tended to be more severe.

**Conclusion:**

Our results suggest that readily available data may help identify children at risk for chronic disease and those whose thrombocytopenia is attributable to a cause other than ITP. Initial treatment decisions appeared to be driven primarily by clinical presentation.

## 1 Introduction

Primary immune thrombocytopenia (ITP) is the most common type of acquired thrombocytopenia in children. It is characterized by severe isolated thrombocytopenia and an increased risk of bleeding (1, 2).

The annual incidence of ITP is estimated at 1.9–6.4/100,000 children, with peak onset between 2 and 5 years of age (1, 3). In early childhood, males are affected more often than females. However, during adolescence, the proportion of affected females approaches or even exceeds that of males (3, 4). In more than half of the cases, ITP onset follows a viral infection or vaccination, leading to seasonal fluctuations in its occurrence (4).

ITP typically presents with cutaneous bleeding. In over 20% of cases, patients may experience more severe symptoms, such as epistaxis, gastrointestinal bleeding, hematuria, or menorrhagia in adolescent females. The most serious complication is intracranial hemorrhage, which has been reported in <0.4% of pediatric patients (5, 6).

Based on disease duration, ITP is classified as newly diagnosed (<3 months), persistent (3–12 months), and chronic (≥12 months) (5). Most children experience a favorable course with spontaneous platelet recovery within the first 3 months. However, in approximately one-quarter of patients, thrombocytopenia persists beyond 12 months (1, 7). Chronic ITP substantially affects children's quality of life and, in addition to raising the risk of bleeding, places a considerable psychological burden on both patients and their families (8, 9).

Research on chronic ITP in children has revealed various risk factors. However, they do not permit reliable prediction of the disease course in everyday clinical practice (10). Owing to the absence of practical predictive tools, the initial management approach tends to be uniform across all children diagnosed with ITP. Yet emerging evidence suggests that transient and chronic forms represent distinct biological and clinical entities, supporting the need for a tailored treatment strategy for the latter (10, 11).

Etiologically, immune thrombocytopenia is classified as primary and secondary forms (2). In primary form, no apparent cause for the decrease in platelet count can be identified (7). Conversely, the secondary form, which is far less common in children, arises from immune dysregulation associated with conditions such as chronic infections and autoimmune diseases, or from the use of certain medications (2, 12).

There is no diagnostic method that can definitively confirm the diagnosis of ITP. Therefore, the diagnosis is made by excluding other potential causes of thrombocytopenia, including secondary immune thrombocytopenia and numerous non-immune (non-IT) disorders, such as inherited thrombocytopenias, malignancies, and coagulopathies (2, 12). According to the current international recommendations, initial diagnostic work-up should focus on a minimal set of investigations—namely, a detailed medical history and clinical examination, a complete blood count, and peripheral blood smear analysis (5, 12). While this streamlined approach helps prevent unnecessary diagnostic procedures, it also increases the risk of delaying the recognition of secondary causes (12). In a small percentage of children who are initially assumed to have ITP, another underlying cause of thrombocytopenia is later identified. Although this percentage is reported to be <3%, it is not negligible, given that the differential diagnoses include conditions requiring urgent recognition and treatment (8, 12).

In ITP, pharmacological therapy is typically reserved for children with clinically significant bleeding, as observation is generally safe even at very low platelet counts. Although first-line options [steroids, intravenous immunoglobulins (IVIg), and anti-D immunoglobulins] can provide short-term benefit, they are associated with adverse effects and substantial costs (8, 13). Because bleeding risk depends on factors beyond platelet count, including comorbidities, social circumstances, and concomitant medications, treatment decisions should consider a comprehensive range of risk factors, which, in the absence of standardized criteria, remain largely at the treating physician's discretion (8, 14).

Overall, the initial presentation of ITP poses several practical challenges related to prognosis, diagnostic certainty, and management. Accordingly, this study aimed to identify clinically relevant factors associated with disease course, later diagnostic revision, and initial management in children newly diagnosed with ITP. Using readily available demographic, clinical, and laboratory data, we evaluated the potential to identify children at increased risk of developing chronic ITP and those with secondary causes of thrombocytopenia. Additionally, we summarized clinical characteristics and outcomes according to initial management strategy. These analyses were intended to provide a more consistent and clinically grounded approach to early care, with the goal of reducing disease burden.

## 2 Materials and methods

### 2.1 Study population

This single-center retrospective study included patients ≤18 years of age who were evaluated for ITP between January 2009 and May 2024 at the University Children's Hospital of the University Medical Center in Ljubljana, Slovenia.

Patients were eligible for inclusion if they met all of the following criteria: (i) they had been assigned an International Classification of Diseases 10th revision (ICD-10) code of D69.3, D69.4, or D69.6; (ii) their platelet count was <150 × 10^9^/L; (iii) their red and white blood cell indices fell within the age-specific reference range, with the exception of patients with microcytic anemia due to iron deficiency or leukopenia/leukocytosis attributable to viral infection; and (iv) they showed no clinical features at presentation that were considered suggestive of an alternative cause of thrombocytopenia (e.g., weight loss, recurrent fever, bone pain, lymphadenopathy, hepatosplenomegaly, positive family history). A platelet count threshold of <150 × 10^9^/L was used because the study period extended across earlier diagnostic definitions of ITP that applied this cutoff. Patients with incomplete clinical documentation, those lost to follow-up, and those with neonatal thrombocytopenia were excluded from the study. A flow diagram illustrating the selection of the study population is provided in the Supplementary Material.

### 2.2 Data collection and definitions

From the hospital's electronic medical records, we collected demographic data, clinical presentation details, laboratory parameters at disease onset and first follow-up blood draw, treatment approaches and adverse effects during both the initial and chronic phases, details on additional investigations performed, bleeding-related complications, follow-up duration, and final outcomes.

A history of infection or vaccination was considered positive if it had occurred within 6 weeks prior to diagnosis. To grade bleeding severity at disease onset and during follow-up, we utilized the modified Buchanan and Adix bleeding score as described by Schoettler et al. (15). Observation was defined as no ITP-directed therapy at the initial presentation. Treatment response was defined as adequate if the platelet count increased to >30 × 10^9^/L, provided there was no clinically significant bleeding (grade ≥3). Bleeding episodes that occurred during follow-up were classified as complications if they reached grade ≥3. Follow-up time was calculated from diagnosis to the last recorded clinical contact due to ITP.

For each patient, we retrospectively determined the final disease outcome. An alternative diagnosis was assigned when thrombocytopenia was attributed to a specific non-ITP etiology based on confirmatory evidence. Because the alternative diagnoses were heterogeneous and individual categories were small, comparative analyses were performed using the combined alternative-diagnosis group. If no alternative etiology was identified, the outcome remained ITP, with patients being further categorized based on the duration of thrombocytopenia as newly diagnosed, persistent, and chronic.

### 2.3 Statistical analysis

Statistical analysis was performed using Microsoft Excel (Version 2506 Build 18925.20158) with the XLSTAT Premium (2025.1.2) add-on. Categorical variables are presented with frequencies and percentages. The distribution of quantitative variables was assessed using the Shapiro–Wilk test. Normally distributed variables are reported as means with standard deviations, whereas non-normally distributed variables are summarized as medians with interquartile ranges. For comparisons of quantitative variables, we employed either a two-sided *t*-test or a Mann–Whitney *U*-test, as appropriate. For comparisons of categorical variables, we utilized the chi-square test or Fisher's exact test with frequencies <5. To evaluate the association between risk factors and the development of chronic ITP, we first performed univariate logistic regression, reporting odds ratios with 95% confidence intervals. Variables that were statistically significant and/or considered clinically important were subsequently entered into a multivariate logistic regression model. We assessed discriminative ability with the area under the receiver operating characteristic curve (ROC AUC), collinearity with variance inflation factors (VIF), and calibration with the Hosmer–Lemeshow test.

For univariate analyses, each variable was analyzed using all patients with available data for that specific variable. Therefore, the number of observations varied across univariate models. To ensure a consistent analytic sample, only variables with complete data were included in the multivariate logistic regression model.

To describe time to first bleeding complication according to initial management strategy, Kaplan–Meier analysis was performed. Time zero was defined as the date of diagnosis. Patients without bleeding complications were censored at the date of last follow-up visit. Complication-free probability was estimated for the pharmacological therapy and watch-and-wait groups.

### 2.4 Ethics

The research was approved by the National Medical Ethics Committee of the Republic of Slovenia (0120-436/2025-2711-3) and conducted in accordance with the Declaration of Helsinki.

## 3 Results

### 3.1 Study population

**Figure 1:**
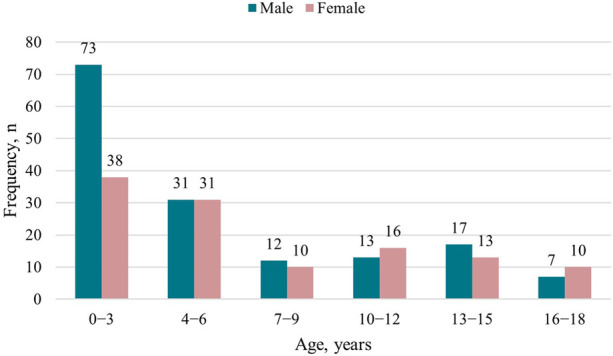
Distribution of patients by age and gender. Bars represent the number of male and female patients within each age group at diagnosis.

A total of 271 patients initially evaluated as ITP were included in the study. Of these, 240 (88.6%) were ultimately diagnosed with ITP, comprising 168 (70.0%) transient and 72 (30.0%) chronic cases. The remaining 31 patients (11.4%) received alternative final diagnoses. Table 1 summarizes patient characteristics, and Figure 1 shows the age and gender distribution.

**Table 1:** Baseline characteristics of the study population.

Characteristic	Children evaluated with a diagnosis of ITP
	*N* = 271
	*n* (%)
Female gender	118 (43.5)
Age (years), median (IQR)	4 (2–10.5)
History of infection or vaccination	195 (72.0)
Platelet count at presentation (×10^9^/L), median (IQR)	10 (3.5; 32.0)
Platelet count <20 × 10^9^/L	180 (66.4)
Clinical presentation at onset (modified Buchanan and Adix bleeding score)	
0	41 (15.1)
1	54 (19.9)
2	81 (29.9)
3a	75 (27.7)
3b	19 (7.0)
4	1 (0.4)
5	0
Initial management	
Observation (watch-and-wait)	142 (52.4)
Upfront therapy (intravenous immunoglobulins, glucocorticoids)	129 (47.6)
Platelet count at first laboratory check after therapy (×10^9^/L); median (IQR)	38 (18.0; 70.3)
Treatment response	78/129 (60.5)
Any adverse effect	65/129 (50.4)
Headache	24/129 (18.6)
Fever	17/129 (13.2)
Dyspnea	1/129 (0.8)
Aseptic meningitis	3/129 (2.3)
Neutropenia (<1,5 × 10^9^/L)	20/129 (15.5)
Treated during chronic phase	32 (11.8)
Bleeding complications during course (modified Buchanan and Adix bleeding score)	39 (14.4)
3a	17/39 (43.6)
3b	19/39 (48.7)
4	1/39 (2.6)
5	2/39 (5.1)

ITP, primary immune thrombocytopenia; IQR, interquartile range.

Twelve patients had baseline platelet counts between 100 and 149 × 10^9^/L. Among them, 2 had a transient clinical course, 9 developed chronic course, and 1 ultimately received an alternative diagnosis. All 9 patients with chronic courses in this platelet-count range were asymptomatic at presentation. The patient with an alternative diagnosis was later diagnosed with inherited macrothrombocytopenia due to a Tubulin beta 1 class IV (TUBB1) mutation.

### 3.2 Transient vs. chronic ITP

Among the 168 patients with transient ITP, 148 (88.1%) had newly diagnosed form and 20 (11.9%) had persistent form. Comparison between the transient and chronic groups is shown in Table 2. The multivariate model demonstrated good discrimination (AUC 0.818) and adequate calibration (Hosmer–Lemeshow *p* = 0.115). All VIF were <2.

**Table 2:** Comparison of transient vs. chronic primary immune thrombocytopenia with univariate and multivariate logistic regression.

Characteristic	Transient ITP	Chronic ITP	Univariate logistic regression				Multivariate logistic regression		
	*N* = 168	*N* = 72					*N* = 240		
	*n* (%)	*n* (%)	*N*	OR	95% CI	*p*-value	OR	95% CI	*p*-value
Male gender	101 (60.1)	36 (50.0)	240	0.66	0.38–1.16	0.148	0.75	0.39–1.45	0.390
Age (years); median (IQR)	3 (2.0; 6.0)	8 (4.0; 13.0)	240	1.15	1.09–1.22	<0.0001	1.08	1.02–1.16	0.017
Bleeding grade <3 at onset (modified Buchanan and Adix bleeding score)	100 (59.5)	51 (70.8)	240	1.55	0.86–2.78	0.148	0.53	0.22–1.26	0.148
History of infection or vaccination	134 (79.8)	42 (58.3)	240	0.36	0.20–0.65	0.001	0.45	0.22–0.92	0.029
Platelet count (×10^9^/L); median (IQR)	6.0 (3.0; 13.0)	32.0 (10.0; 64.5)	240	1.04	1.03–1.05	<0.0001	1.03	1.02–1.05	<0.0001
MPV (fL); median (IQR)	9.6 (8.8; 10.8)	11.3 (10.4; 12.4)	95	1.15	0.95–1.40	0.160			
Leukocytes (×10^9^/L); median (IQR)	8.9 (7.3; 11.3)	7.1 (5.5; 8.9)	207	0.80	0.70–0.90	0.0004			
Hemoglobin (g/L); median (IQR)	121.5 (114.8; 127.0)	128.0 (118.5; 134.3)	208	1.01	0.99–1.04	0.182			
MCV (fL); mean ± SD	76.9 ± 4.6	78.0 ± 5.1							
Neutrophils (×10^9^/L); median (IQR)	3.5 (2.5; 4.5)	3.1 (2.0; 3.8)							
Lymphocytes (×10^9^/L); median (IQR)	4.2 (2.7; 6.0)	2.7 (2.1; 3.4)	172	0.65	0.51–0.83	0.0005			
Monocytes (×10^9^/L); median (IQR)	0.5 (0.4; 0.8)	0.5 (0.4; 0.7)							
Eosinophils (×10^9^/L); median (IQR)	0.2 (0.1; 0.3)	0.1 (0.1; 0.2)							
Treated	97 (57.7)	20 (27.8)	240	0.28	0.15–0.51	<0.0001	0.59	0.24–1.46	0.256
Treatment response	62/97 (63.9)	14 (70.0)	116	1.52	0.53–4.76	0.415			

ITP, primary immune thrombocytopenia; OR, odds ratio; CI, confidence interval; IQR, interquartile range; MPV, mean platelet volume; MCV, mean corpuscular volume; SD, standard deviation.

### 3.3 Alternative diagnoses

**Figure 2:**
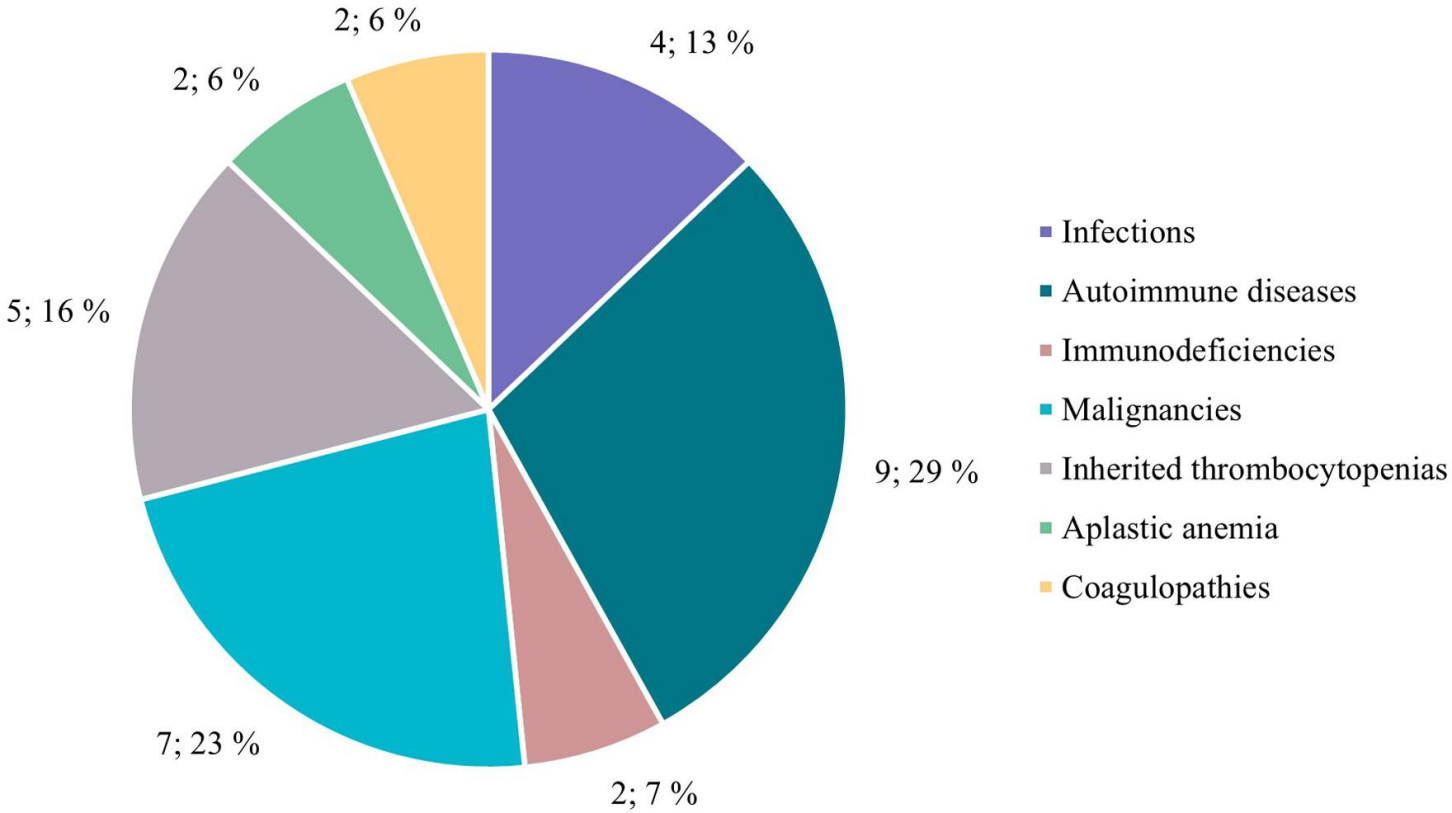
Causes of secondary immune thrombocytopenia and non-immune thrombocytopenia. Counts are shown as *n* (%): Infections: *n* = 4, *H. pylori*. Autoimmune diseases: *n* = 1, antiphospholipid syndrome; *n* = 3, Evans syndrome; *n* = 4, systemic lupus erythematosus; *n* = 1, autoimmune pancytopenia. Immunodeficiencies: *n* = 1, combined immunodeficiency [IKAROS family zinc finger 1 (IKZF1) mutation]; *n* = 1, DiGeorge syndrome. Malignancies: *n* = 5, myelodysplastic syndrome; *n* = 1, juvenile myelomonocytic leukemia; *n* = 1, Hodgkin lymphoma. Inherited thrombocytopenias: *n* = 2, macrothrombocytopenia [Tubulin beta 1 class IV (TUBB1) and Actinin alpha 1 (ACTN1) mutations]; *n* = 1, RUNX family transcription factor 1 (RUNX1)-associated thrombocytopenia; *n* = 1, Wiskott-Aldrich syndrome [Actin related protein 2/3 complex subunit 1B (ARPC1B) deficiency]; *n* = 1, congenital amegakaryocytic thrombocytopenia. Coagulopathies: *n* = 1, congenital hypofibrinogenemia [Fibrinogen gamma chain (FGG) mutation]; *n* = 1, kaposiform hemangioendothelioma with Kasabach–Merritt phenomenon.

An alternative cause of thrombocytopenia was identified in 31 patients. Figure 2 displays the distribution of these alternative diagnoses categorized by disease type. Individual final diagnoses, baseline platelet counts, bleeding phenotypes, age, and confirmatory tests are presented in Table 3. Patient diagnosed with DiGeorge syndrome was also confirmed to have a *H. pylori* infection. A comparison between patients with ITP and those with alternative diagnoses is presented in Table 4.

**Table 3:** Final diagnoses, baseline characteristics, and confirmatory tests of patients with alternative diagnoses.

Final diagnosis	*N*	Baseline platelet count (×10^9^/L), median (range)	Bleeding phenotype at onset	Age (years), median (range)	Confirmatory tests
*Helicobacter pylori*-associated thrombocytopenia	4	10 (0–30)	Heterogeneous; grades 1, 2, 3a, and 4	11 (11–15)	*H. pylori* fecal antigen test
Systemic lupus erythematosus	4	17 (3–56)	Cutaneous only; grades 1–2	10 (3–14)	Autoimmune serology (ANA, anti-dsDNA)
Evans syndrome	3	3 (1–4)	Heterogeneous; grades 0, 1, and 3a	5 (0–17)	Direct Coombs test
Antiphospholipid syndrome	1	4	Grade 3b	15	Antiphospholipid antibody testing
Autoimmune pancytopenia	1	7	Grade 2	9	Antiplatelet antibodies, antineutrophil antibodies, direct Coombs test
Immunodeficiencies	2	31.5 (3–60)	Heterogeneous, grades 1 and 3a	7 (0–14)	Molecular genetic testing
Myelodysplastic syndrome	5	69 (29–78)	No bleeding; grade 0	12 (1–15)	Bone marrow aspiration
Hodgkin lymphoma	1	12	Grade 2	11	Lymph node fine-needle aspiration
Juvenile myelomonocytic leukemia	1	30	Grade 1	1	Bone marrow aspiration
Inherited thrombocytopenia	5	36 (5–115)	No bleeding or cutaneous only; grades 0–1	4 (0–16)	Molecular genetic testing
Aplastic anemia	2	49.5 (46–53)	No bleeding; grade 0	7 (1–13)	Bone marrow aspiration
Coagulopathies	2	17.5 (3–32)	Cutaneous and mild mucosal bleeding; grades 2–3a	4 (4–4)	Coagulation studies

Continuous variables are presented as median (range). For diagnoses represented by a single patient, individual values are shown. Bleeding phenotype at onset was classified according to the modified Buchanan and Adix bleeding score. ANA, antinuclear antibodies; anti-dsDNA, anti-double-stranded DNA antibodies.

**Table 4:** Comparison of patients with primary immune thrombocytopenia vs. patients with an alternative cause of thrombocytopenia.

Characteristic	ITP	Alternative causes (secondary ITP and non-IT)	*p*-value
	*N* = 240	*N* = 31	
	*n* (%)	*n* (%)	
Female gender	103 (42.9)	15 (48.4)	0.563
Age (years), median (IQR)	4 (2.0; 9.3)	11 (3.5; 13.0)	0.034
Clinical presentation at onset (modified Buchanan and Adix bleeding score)			
0	30 (12.5)	11 (35.5)	0.037^a^
1	46 (19.2)	8 (25.8)	
2	75 (31.3)	6 (19.4)	
3a	71 (29.6)	4 (12.9)	
3b	18 (7.5)	1 (3.2)	
4	0	1 (3.2)	
History of infection or vaccination	176 (73.3)	19 (61.3)	0.160
Platelet count (×10^9^/L), median (IQR)	9.0 (3.0; 28.0)	29.0 (4.0; 52.0)	0.067
MPV (fL), median (IQR)	10.0 (8.9; 11.3)	10.2 (9.5; 11.3)	0.635
Leukocytes (×10^9^/L), median (IQR)	8.6 (6.7; 10.6)	6.4 (5.2; 8.3)	0.002
Hemoglobin (g/L), median (IQR)	124.0 (115.0; 130.0)	121.0 (113.0; 131.5)	0.691
MCV (fL), median (IQR)	77.3 (74.2; 80.0)	79.9 (77.0; 86.5)	0.001
Neutrophils (×10^9^/L), median (IQR)	3.4 (2.3; 4.4)	3.4 (2.6; 3.7)	0.862
Lymphocytes (×10^9^/L), median (IQR)	3.6 (2.5; 5.3)	2.5 (2.0; 3.3)	0.010
Monocytes (×10^9^/L), median (IQR)	0.5 (0.4; 0.8)	0.3 (0.2; 0.6)	0.011
Eosinophils (×10^9^/L), median (IQR)	0.2 (0.1; 0.3)	0.1 (0.0; 0.1)	0.014

ITP, primary immune thrombocytopenia; non-IT, non-immune thrombocytopenia; IQR, interquartile range; MPV, mean platelet volume; MCV, mean corpuscular volume.

a Monte Carlo method.

Additional diagnostic tests were performed in 193/271 patients (71.2%). *Helicobacter pylori* testing was positive in 5/24 (20.8%), antinuclear antibodies in 9/49 (18.4%), and the direct Coombs test in 9/35 (25.7%). Molecular genetic testing yielded (likely) pathogenic variants in 13/32 (40.6%). Bone marrow aspiration revealed pathological findings in 3/18 patients (16.7%) at onset and in 7/31 (22.6%) during chronic phase. Antiplatelet antibodies were positive in 14/66 (21.2%) of ITP patients and 10/19 (52.6%) of those with an alternative diagnosis (*p* = 0.007).

### 3.4 Treatment

Among 129 treated patients, 122 (94.6%) received IVIg alone, and 7 (5.4%) received combination therapy with IVIg and glucocorticoids. None of the patients received anti-D immunoglobulins. At disease onset, 32 (11.8%) patients received supportive treatment, administered either as standalone therapy or in addition to upfront pharmacological treatment. Of these, 2 (6.3%) were managed with local tamponade for epistaxis, 23 (71.9%) received tranexamic acid, 4 (12.5%) received platelet transfusions, 2 (6.3%) received red blood cell transfusions, and 1 (3.1%) received combination of vitamin K and eltrombopag. Regarding adverse effects, all patients who experienced treatment-related adverse effects had received IVIg therapy, including 2 patients who received IVIg in combination with glucocorticoids.

In the ITP group, 123 patients (51.3%) were managed with observation, and 117 (48.8%) received upfront pharmacological therapy. Patient characteristics according to initial management strategy are presented in Table 5.

**Table 5:** Characteristics of patients with primary immune thrombocytopenia according to management strategy.

Characteristic	Pharmacological therapy	Watch-and-wait strategy
	*N* = 117	*N* = 123
	*n* (%)	*n* (%)
Female gender	51 (43.6)	52 (42.3)
Age (years), median (IQR)	3.0 (1.0; 6.0)	6.0 (2.0; 12.0)
Bleeding grade ≥3 at onset (Buchanan and Adix bleeding score)	76 (65.0)	13 (10.6)^a^
Platelet count (×10^9^/L), median (IQR)	4.0 (3.0; 7.0)	24.0 (10.0; 52.2)
Platelet count <20 × 10^9^/L	109 (93.2)	56 (45.5)
Complications (Buchanan and Adix bleeding score ≥3)	22 (18.8)	13 (10.6)
Follow-up duration (months), median (IQR)	3 (1; 12)	10 (1; 37.5)

IQR, interquartile range.

a Among the 13 patients with bleeding grade ≥3 at onset, 5 patients had platelet counts <20 × 10^9^/L.

In a subgroup of treated patients with platelet counts <20 × 10^9^/L, 38/109 (34.9%) had a bleeding score <3, indicating absent or cutaneous bleeding only, compared with 51/56 observed patients (91.1%) with platelet counts <20 × 10^9^/L.

**Figure 3:**
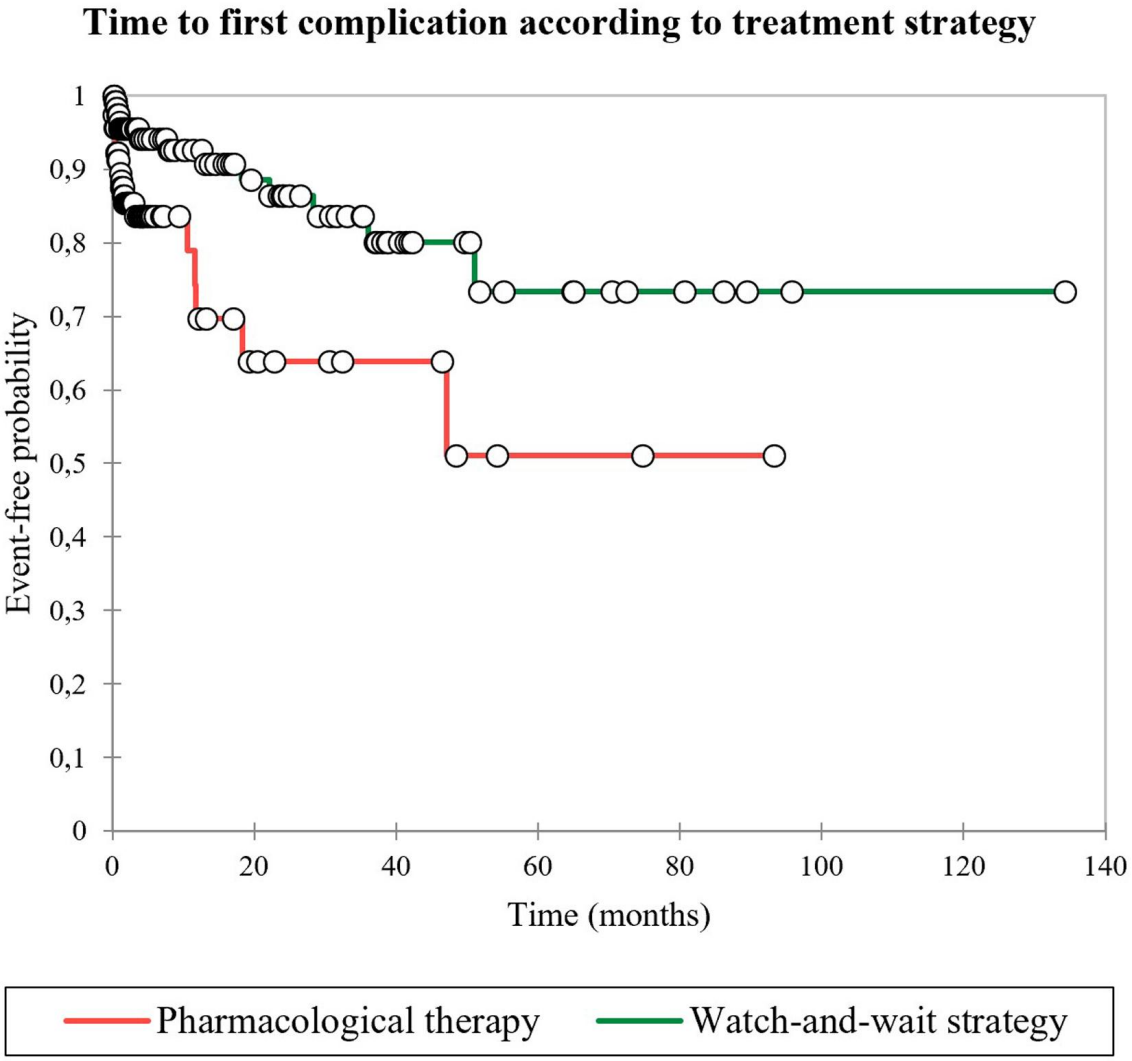
Kaplan–Meier curve showing complication-free probability according to initial management strategy. The event was defined as the first bleeding complication with Buchanan and Adix bleeding grade ≥3. Patients without bleeding complications were censored at the date of last follow-up visit.

In the treated group, 22/117 patients (18.8%) experienced bleeding complications, 12 graded as 3a and 10 as 3b. In the observation group, 13/123 patients (10.6%) experienced bleeding complications: 5 graded as 3a and 6 as 3b, while one patient each had grade 4 and grade 5 bleeding. The risk difference between the groups was +8.2% (95% CI −0.7% to 17.2%). The Kaplan–Meier curve is presented in Figure 3.

## 4 Discussion

This retrospective single-center study analyzed children managed as ITP over a 15-year period. Its aim was to examine factors associated with chronic ITP, characterize alternative causes of thrombocytopenia among children initially managed as ITP, and evaluate the initial management approach. Our findings suggest that routinely available data may help identify children at risk for developing chronic ITP and those in whom thrombocytopenia is more likely attributable to an alternative cause, while treatment decisions appeared to be driven primarily by clinical presentation. These findings add valuable regional evidence to the pediatric ITP literature.

The proportion of patients with chronic ITP in our cohort appeared to be higher than that reported previously, reaching 30% (16). This difference likely reflects our methodology, as we included all patients evaluated under a diagnosis of ITP from 2009 onward, even if the initial diagnosis had been established earlier. This approach may have enriched the cohort for patients with chronic courses. In addition, the higher proportion may partly reflect a deeper understanding and more consistent application of current criteria for chronic ITP.

Consistently reported risk factors for chronic ITP include older age, female gender, the absence of preceding infection or vaccination, higher platelet count at diagnosis, and an insidious onset (10, 17). In our multivariate analysis, we found that older age, a higher platelet count at diagnosis, and a lack of a preceding infection or vaccination were independently associated with chronicity. Additional variables were significant in univariate analyses but lost significance after adjustment or could not be evaluated due to missing data. Their potential associations warrant further investigation in future studies.

The presence of immunological triggering factors, such as viral antigens or antigens introduced through active immunization, supports the hypothesis that cross-reactivity between exogenous antigens and platelets contributes to the initiation of ITP. This process leads to the generation of autoantibodies and subsequent enhanced peripheral platelet destruction (11, 18). In transient ITP, this priming phase presumably subsides after elimination of the triggering antigen, resulting in spontaneous recovery of platelet counts. In contrast, in patients who develop chronic ITP, additional factors, such as a pre-existing inflammatory milieu, impaired immune tolerance, or persisting antigenemia, may facilitate the establishment of an immune memory response, which is more challenging to terminate (11).

Although the hypothesis mentioned supports the idea that immunological triggers play a role in the initiation of ITP, it does not explain why this factor appears to differ between transient and chronic disease. Both forms are believed to follow immune stimulation; however, in transient ITP, the immune response eventually diminishes, whereas in chronic ITP, it persists. One possible explanation for this discrepancy relates to timing, as the establishment and stabilization of memory response require time. The time lag between immune stimulation and clinical expression may therefore exceed the window captured by routine history-taking or arbitrarily defined timeframes used in studies (e.g., 6 weeks in our study) (10, 11).

The independent association between a higher initial platelet count and chronic ITP may point to the role of platelet burden in establishing and maintaining immune memory response (11, 17, 19). Epitope spreading offers one conceptual framework, whereby the autoantibody response broadens from initial to additional platelet autoantigens, resulting in a wider spectrum of autoantibodies (19, 20). A higher platelet count, particularly when paired with an increased mean platelet volume (MPV), could provide a larger pool of potential immune targets available for humoral response expansion (19). Furthermore, it is warranted to hypothesize that higher platelet counts may contribute to disease persistence through platelet-mediated immunomodulatory effects, which remain insufficiently defined and require further research (21, 22).

Contrary to expectations, the proportion of females in the chronic group did not stand out and accounted for 50% (10, 17). Although there is no well-founded explanation for this observation, the increase in the proportion of females among older age groups, which coincides with hormonal maturation, at least partly supports the established hypothesis regarding the role of female gender in the development of autoimmune diseases (23).

Finally, we did not observe an independent association between treatment and disease course. Although some previous studies have reported this association, this finding is not entirely unexpected, as the apparent treatment effect may be explained by other variables included in the multivariate analysis (16, 24). Given that appropriate treatment could, at least theoretically, interrupt the priming phase of the immune response and thereby reduce the likelihood of establishing a durable immune response, further studies are needed to more precisely define the role of initial treatment in the course of ITP (11).

The proportion of patients in whom the initial diagnosis of ITP was revised to an alternative diagnosis was 11.4%. This is substantially higher than in previous studies and may reflect our single-center tertiary-care setting (12).

When thrombocytopenia initially labeled as ITP was later reassigned to an alternative diagnosis, patients exhibited a distinct profile: they were older at diagnosis, with lower bleeding grade, lower leukocyte differential counts, and higher mean corpuscular volume (MCV). In clinical practice, such features are commonly regarded as “red flags” prompting broader evaluation (12).

Higher lymphocyte, monocyte, and eosinophil counts together accounted for the higher total leukocyte count. This finding is consistent with current understanding of the immunological mechanisms involved in ITP. During peripheral platelet destruction, T-cells and B-cells are activated and proliferate. Monocytes may further support and sustain immune responses at multiple levels, including secretion of B-cell activating factor, differentiation into dendritic cells, and downstream pathological stimulation of T-cells (25).

An elevated MCV may provide an important laboratory clue to bone marrow or nutritional disorders that require timely recognition and treatment. However, in thrombocytopenic patients with bleeding, interpretation of red cell indices may be challenging. Reticulocytosis following acute hemorrhage can increase MCV, whereas prolonged or unrecognized blood loss with iron deficiency may decrease it, potentially obscuring an underlying MCV abnormality (26, 27). Notably, silent bleeding has been described in children with ITP at several sites, including urinary and gastrointestinal tracts, where blood loss may be clinically relevant, as well as retina and central nervous system. Such bleeding may not be apparent from history or physical examination alone, or may present only with subtle clinical signs, such as behavioral changes in the case of brain microbleeds. Further studies are needed to better define its potential contribution to iron deficiency anemia and the role of targeted diagnostic and supportive strategies (28). To conclude, MCV should be interpreted cautiously and always in conjunction with the broader clinical context and complementary laboratory findings, while considering the possibility of silent bleeding.

Because the inclusion criterion was age-appropriate red and white blood cell indices, these laboratory parameters remained within the reference range. Nevertheless, such data contains clinically relevant information. Subtle shifts and complex patterns may not be readily apparent to clinicians or captured by simple rule-based approaches, but can be detected by machine-learning methods. In other clinical settings, these approaches have demonstrated high diagnostic performance and may serve as a valuable adjunct to established diagnostic pathways (29).

A comparison between the ITP group and a combined group of secondary thrombocytopenias can provide a rough overview of characteristics associated with ITP and point to red flags. However, this approach is suboptimal because the comparator group comprises a highly heterogeneous set of disorders. Pooling such disparate entities may obscure clinically important differences and lead to misleading conclusions.

Diagnosis-level assessment suggested that immune-mediated and infectious causes more closely resembled ITP at presentation, whereas etiologically distinct disorders, including bone marrow failure, clonal hematologic disorders, and inherited thrombocytopenias, showed more divergent baseline features, such as higher baseline platelet counts and milder bleeding phenotypes. However, interpretation is limited by the small number of patients within individual diagnostic subgroups.

When and how far to extend the initial diagnostic work-up to exclude secondary causes without exposing patients to unnecessary testing remains an open question. In our cohort, >20% of tested patients were *H. pylori*–positive, indicating that a meaningful proportion of children had a potentially treatable infectious finding. This is clinically important, as *H. pylori* can be detected using non-invasive methods and eradication therapy is widely available, affordable, and generally well tolerated. Accordingly, *H. pylori* testing represents one of the few readily actionable additions to the diagnostic pathway, although its impact on platelet recovery remains uncertain (8, 30).

Current guidance, therefore, remains nuanced. In adults, international consensus recommendations suggest incorporating *H. pylori* testing in selected geographic regions, whereas in children, it has generally been reserved for those with persistent thrombocytopenia (8). However, the most recent ESPGHAN/NASPGHAN guidelines do not recommend routine *H. pylori* testing even in chronic ITP, reflecting heterogeneity in the pediatric literature (31). At the same time, local epidemiology may influence the risk-benefit balance. Slovenian data suggest that Cytotoxin-associated antigen A (CagA)–positive strains, reported to be associated with a higher likelihood of platelet response after eradication, are present in >60% of pediatric *H. pylori* infections (32). Collectively, these observations suggest that Slovenia, and countries with similar epidemiology, may represent settings in which earlier incorporation of H. pylori testing warrants consideration.

Antiplatelet antibodies were detected more frequently in children with thrombocytopenia due to causes other than ITP, highlighting potential overlap in immune-mediated mechanisms and poor diagnostic value of the assays used. Accordingly, routine platelet antibody testing is not recommended for the diagnosis of ITP (8). Nevertheless, growing evidence suggests that antigen-specific antibody profiling may improve disease phenotyping, as distinct platelet targets have been associated with differences in treatment response and disease course, potentially enabling more individualized management (33).

In our cohort, pharmacologically treated and observed patients were represented in similar proportions, suggesting a relatively low threshold for initiating therapy, given reports that up to 84% of newly diagnosed pediatric ITP cases could be safely observed (8, 13). Higher treatment rates also increase exposure to treatment-related adverse effects, which were generally mild in our cohort (most commonly fever, headache, and neutropenia), but still prompted additional medical interventions and prolonged hospitalizations.

Overall, our findings indicate that management decisions were guided primarily, but not exclusively, by the severity of clinical presentation. Notably, approximately one-third of treated patients presented with low-grade bleeding at onset, suggesting that treatment was initiated in the subset of patients on the basis of broader clinical judgement, including severe thrombocytopenia, platelet count trajectory, caregiver understanding, access to urgent reassessment, and social circumstances affecting domestic safety. Age appeared to be an important factor in treatment selection, as treated patients were younger than those managed with observation. However, this difference may reflect greater caution in the management of younger children, age-related differences in disease characteristics, or both.

Conversely, severe thrombocytopenia was also common among observed patients, with nearly half having platelet counts <20 × 10^9^/L, yet most had no bleeding or cutaneous bleeding only, corresponding to bleeding grade <3. This reflects guideline-based practice, in which pharmacological therapy is generally reserved for clinically relevant bleeding rather than platelet count alone (8). Thus, observation was considered appropriate in patients with severe thrombocytopenia but absent or mild bleeding, provided that close monitoring was feasible. In selected cases of mild mucosal bleeding, most commonly mild epistaxis or menorrhagia, local interventions or hemostatic agents were used as temporary supportive strategies, together with clear instructions to seek urgent reassessment if bleeding worsened.

The discordance between platelet count and bleeding severity supports the view that platelet count alone incompletely captures bleeding risk and that bleeding phenotypes vary substantially due to additional hemostatic determinants (16, 34). Accordingly, ITP is increasingly viewed as a disorder of global hemostasis, in which increased basal platelet activation and downstream coagulation activation may partially preserve effective hemostasis despite profound thrombocytopenia (35). Taken together, these findings are consistent with an approach in which treatment decisions are guided primarily by clinical presentation and raise the possibility that, in a subset of patients who require therapy, interventions addressing a broader range of hemostatic disturbances beyond predominantly immune-mediated mechanisms could be beneficial.

Within the evolving treatment framework, thrombopoietin receptor agonists (TPO-RAs), such as eltrombopag and romiplostim, are being explored beyond their traditional role in chronic ITP. In the PINES randomized clinical trial, eltrombopag improved sustained platelet response compared with standard first-line pharmacological therapy in newly diagnosed pediatric ITP, supporting further evaluation of TPO-RAs in early disease management (36).

Complications were summarized according to initial management strategy. They were more frequent among patients receiving pharmacological therapy than among those managed with watch-and-wait, whereas complications in the watch-and-wait group tended to be more severe. Kaplan–Meier analysis was used to visualize time to first bleeding complication across management groups, accounting for differences in follow-up duration. Complication-free probability appeared lower among patients receiving pharmacological therapy than among those managed with a watch-and-wait strategy. As treatment allocation was not randomized and baseline characteristics differed substantially between groups, the analysis should be interpreted descriptively. Prospective studies with appropriate adjustment for baseline disease severity are needed to more reliably assess treatment safety and effectiveness.

## 5 Limitations

Limitations of this study include its retrospective design, as data capture depended on clinicians' documentation and clinical judgment, despite predefined study definitions. Standardization was further limited by changes in disease definitions over the study period, and some older cases lacked complete data. As a single-center study, our findings may also reflect local referral patterns and practice characteristics, which may limit generalizability.

## 6 Conclusions

In conclusion, our findings suggest that children at increased risk of chronic ITP and those with thrombocytopenia due to alternative causes may be identifiable using data from the initial diagnostic evaluation. In our cohort, treatment decisions were largely associated with clinical severity. With 271 children included, this cohort provides a valuable basis for future studies aimed at refining risk stratification and diagnostic pathways.

## Data Availability

The raw data supporting the conclusions of this article will be made available by the authors, without undue reservation.

## References

[1] Terrell DR Beebe LA Vesely SK Neas BR Segal JB George JN. The incidence of immune thrombocytopenic purpura in children and adults: a critical review of published reports. Am J Hematol. (2010) 85(3):174–80. 10.1002/ajh.21616 20131303

[2] Rodeghiero F Stasi R Gernsheimer T Michel M Provan D Arnold DM. Standardization of terminology, definitions and outcome criteria in immune thrombocytopenic purpura of adults and children: report from an international working group. Blood. (2009) 113(11):2386–93. 10.1182/blood-2008-07-162503 19005182

[3] Yong M Schoonen WM Li L Kanas G Coalson J Mowat F. Epidemiology of paediatric immune thrombocytopenia in the general practice research database. Br J Haematol. (2010) 149(6):855–64. 10.1111/j.1365-2141.2010.08176.x 20377590

[4] Zeller B Rajantie J Hedlund-Treutiger I Tedgård U Wesenberg F Jonsson OG. Childhood idiopathic thrombocytopenic purpura in the nordic countries: epidemiology and predictors of chronic disease. Acta Paediatr Oslo Nor. (2005) 94(2):178–84. 10.1111/j.1651-2227.2005.tb01887.x 15981751

[5] Neunert C Terrell DR Arnold DM Buchanan G Cines DB Cooper N. American Society of Hematology 2019 guidelines for immune thrombocytopenia. Blood Adv. (2019) 3(23):3829–66. 10.1182/bloodadvances.2019000966 31794604 PMC6963252

[6] Pietras NM Gupta N Justiz Vaillant AA Pearson-Shaver AL. Immune thrombocytopenia. In: StatPearls [Internet]. Treasure Island (FL): StatPearls Publishing (2025). Available online at: https://www.ncbi.nlm.nih.gov/books/NBK562282/ (Accessed June 25, 2025). 32965953

[7] Friedman JN Beck CE. Diagnosis and management of typical, newly diagnosed primary immune thrombocytopenia (ITP) of childhood. Paediatr Child Health. (2019) 24(1):54–5. 10.1093/pch/pxy197 30833822 PMC6376287

[8] Provan D Arnold DM Bussel JB Chong BH Cooper N Gernsheimer T. Updated international consensus report on the investigation and management of primary immune thrombocytopenia. Blood Adv. (2019) 3(22):3780–817. 10.1182/bloodadvances.2019000812 31770441 PMC6880896

[9] Schifferli A. Immune thrombocytopenia in adolescents and young adults. Front Med. (2025) 12:1553936. 10.3389/fmed.2025.1553936 PMC11979193 40206467

[10] Heitink-Pollé KMJ Nijsten J Boonacker CWB de Haas M Bruin MCA. Clinical and laboratory predictors of chronic immune thrombocytopenia in children: a systematic review and meta-analysis. Blood. (2014) 124(22):3295–307. 10.1182/blood-2014-04-570127 25305206

[11] Cuker A Prak E Cines D. Can immune thrombocytopenia be cured with medical therapy? Semin Thromb Hemost. (2015) 41(4):395–404. 10.1055/s-0034-1544001 25793364 PMC12880612

[12] Schifferli A Heiri A Imbach P Holzhauer S Seidel MG Nugent D. Misdiagnosed thrombocytopenia in children and adolescents: analysis of the pediatric and adult registry on chronic ITP. Blood Adv. (2021) 5(6):1617–26. 10.1182/bloodadvances.2020003004 33710335 PMC7993109

[13] Cooper N Cines DB. The child with immune thrombocytopenia: to treat or not to treat, is that still the question? Haematologica. (2019) 104(11):2132–4. 10.3324/haematol.2019.229179 31666343 PMC6821618

[14] Russo G Parodi E Farruggia P Notarangelo LD Perrotta S Casale M. Recommendations for the management of acute immune thrombocytopenia in children. A consensus conference from the Italian Association of Pediatric Hematology and Oncology: AIEOP consensus for childhood acute ITP. Blood Transfus. (2024) 22(3):253–65. 10.2450/BloodTransfus.501 37677093 PMC11073630

[15] Schoettler ML Graham D Tao W Stack M Shu E Kerr L. Increasing observation rates in low-risk pediatric immune thrombocytopenia using a standardized clinical assessment and management plan (SCAMP®). Pediatr Blood Cancer. (2017) 64(5):e26303. 10.1002/pbc.26303 PMC5366076 27781392

[16] Heitink-Pollé KMJ Uiterwaal CSPM Porcelijn L Tamminga RYJ Smiers FJ van Woerden NL. Intravenous immunoglobulin vs observation in childhood immune thrombocytopenia: a randomized controlled trial. Blood. (2018) 132(9):883–91. 10.1182/blood-2018-02-830844 29945954

[17] Grimaldi-Bensouda L Nordon C Leblanc T Abenhaim L Allali S Armari-Alla C. Childhood immune thrombocytopenia: a nationwide cohort study on condition management and outcomes. Pediatr Blood Cancer. (2017) 64(7):e26389. 10.1002/pbc.26389 27905681

[18] Semple JW Schifferli A Cooper N Saad H Mytych DT Chea LS. Immune thrombocytopenia: pathophysiology and impacts of romiplostim treatment. Blood Rev. (2024) 67:101222. 10.1016/j.blre.2024.101222 38942688

[19] Jaime-Pérez JC Ramos-Dávila EM Meléndez-Flores JD Gómez-De León A Gómez-Almaguer D. Insights on chronic immune thrombocytopenia pathogenesis: a bench to bedside update. Blood Rev. (2021) 49:100827. 10.1016/j.blre.2021.100827 33771403

[20] Al-Samkari H Rosovsky RP Karp Leaf RS Smith DB Goodarzi K Fogerty AE. A modern reassessment of glycoprotein-specific direct platelet autoantibody testing in immune thrombocytopenia. Blood Adv. (2020) 4(1):9–18. 10.1182/bloodadvances.2019000868 31891657 PMC6960466

[21] Sonmez O Sonmez M. Role of platelets in immune system and inflammation. Porto Biomed J. (2017) 2(6):311–4. 10.1016/j.pbj.2017.05.005 32258788 PMC6806752

[22] Maouia A Rebetz J Kapur R Semple JW. The immune nature of platelets revisited. Transfus Med Rev. (2020) 34(4):209–20. 10.1016/j.tmrv.2020.09.005 33051111 PMC7501063

[23] Song Y Li J Wu Y. Evolving understanding of autoimmune mechanisms and new therapeutic strategies of autoimmune disorders. Signal Transduct Target Ther. (2024) 9(1):263. 10.1038/s41392-024-01952-8 39362875 PMC11452214

[24] Tamminga R Berchtold W Bruin M Buchanan GR Kühne T. Possible lower rate of chronic ITP after IVIG for acute childhood ITP an analysis from registry I of the intercontinental cooperative ITP study group (ICIS). Br J Haematol. (2009) 146(2):180–4. 10.1111/j.1365-2141.2009.07743.x 19466971

[25] Mititelu A Onisâi MC Roșca A Vlădăreanu AM. Current understanding of immune thrombocytopenia: a review of pathogenesis and treatment options. Int J Mol Sci. (2024) 25(4):4. 10.3390/ijms25042163 PMC10889445 38396839

[26] Bonadies N Rovó A Porret N Bacher U. When should we think of myelodysplasia or bone marrow failure in a thrombocytopenic patient? A practical approach to diagnosis. J Clin Med. (2021) 10(5):1026. 10.3390/jcm10051026 33801484 PMC7958851

[27] Green R Dwyre DM. Evaluation of macrocytic anemias. Semin Hematol. (2015) 52(4):279–86. 10.1053/j.seminhematol.2015.06.001 26404440

[28] Tantawy AA Elsherif NHK Kenny MA Aboulfotouh KA Hassan AE Kabil ME. Silent bleeding in children and adolescents with immune thrombocytopenia: relation to laboratory parameters and health related quality of life. J Thromb Thrombolysis. (2020) 50(2):258–66. 10.1007/s11239-020-02036-4 31956939

[29] Podnar S Kukar M Gunčar G Notar M Gošnjak N Notar M. Diagnosing brain tumours by routine blood tests using machine learning. Sci Rep. (2019) 9:14481. 10.1038/s41598-019-51147-3 31597942 PMC6785553

[30] Stasi R Sarpatwari A Segal JB Osborn J Evangelista ML Cooper N. Effects of eradication of Helicobacter pylori infection in patients with immune thrombocytopenic purpura: a systematic review. Blood. (2009) 113(6):1231–40. 10.1182/blood-2008-07-167155 18945961

[31] Homan M Jones NL Bontems P Carroll MW Czinn SJ Gold BD. Updated joint ESPGHAN/NASPGHAN guidelines for management of Helicobacter pylori infection in children and adolescents (2023). J Pediatr Gastroenterol Nutr. (2024) 79(3):758–85. 10.1002/jpn3.12314 39148213

[32] Homan M. Obravnava otrok, okuženih z bakterijo Helicobacter pylori. Zdrav Vestn. (2018) 87(7–8):378–84. 10.6016/ZdravVestn.2646

[33] Grundner M Rožman P Skopec B. Določanje trombocitnih protiteles pri diagnosticiranju imunske trombocitopenije. Slov Med J. (2024) 93(11–12):421–7. 10.6016/ZdravVestn.3546

[34] Almazni I Stapley R Morgan NV. Inherited thrombocytopenia: update on genes and genetic variants which may be associated with bleeding. Front Cardiovasc Med. (2019) 6:80. 10.3389/fcvm.2019.00080 31275945 PMC6593073

[35] Pincez T Mathews N Bonnefoy A. Haemostasis alterations in immune thrombocytopenia and their clinical significance. Res Pract Thromb Haemost. (2025) 9(7):103210. 10.1016/j.rpth.2025.103210 41216430 PMC12597007

[36] Shimano KA Grimes AB Kaicker S Shah SJ Gunn E Bhat RV. Eltrombopag for newly diagnosed pediatric immune thrombocytopenia requiring treatment: the PINES randomized clinical trial. J Am Med Assoc. (2025) 334(20):1816–26. 10.1001/jama.2025.18168 PMC12547676 41123939

