# Supplementary material for: Fifteen years of pediatric immune thrombocytopenia in a national cohort: chronicity, diagnostic challenges, and treatment patterns—single center experience
Source: Front Pediatr. 2026 Jun 11;14:1864433. doi: 10.3389/fped.2026.1864433 (PMC13294345; doi:10.3389/fped.2026.1864433)
Supplement: Supplementary file 1 [file Supplementaryfile1.docx]

Supplementary Material

Flow diagram 1: Selection of the study population.

**Patient enrollment:**

1. University Children’s Hospital of the University Medical Center in Ljubljana, Slovenia,
2. January 2009−May 2024,
3. <19 years of age,
4. ICD-10 codes: D69.3, D69.4, and D69.6.

**N=325**

**Confirmation of case eligibility based on fulfillment of inclusion criteria:**

1. Platelet count <150×10⁹/L,
2. red and white blood cell indices within the age-specific reference range,
3. no clinical features suggestive of a secondary cause of thrombocytopenia.

**N=302**

**Analysis**

**N=271**

**Exclusion:**

1. Patients with incomplete documentation in the electronic medical records or those lost to follow-up. **N=29**
2. Patients with neonatal thrombocytopenia. **N=2**
